# All-cause health-adjusted life expectancy in Jiangxi Province, China, 2000–2030: systematic estimation and analysis

**DOI:** 10.1016/j.pmedr.2026.103436

**Published:** 2026-02-28

**Authors:** Jing Ding, Sanqian Chen, Yuting Deng, Siyu Zhou, Zhang Zeng, Songbo Hu

**Affiliations:** a School of Public Health, Jiangxi Medical College, Jiangxi Provincial Key Laboratory of Disease Prevention and Public Health, Nanchang University, Donghu Campus, Nanchang, Nanchang 330006, PR China; b School of Public Health, Nanchang Medical College, Nanchang 330052, PR China; c Department of Public Health Management Section, Jiujiang City Key Laboratory of Cell Therapy, The First Hospital of Jiujiang City, Jiujiang 332000, PR China

**Keywords:** Model application, All-cause years lived with disability calculation model, All-cause healthy-adjusted life expectancy, Projection, Jiangxi Province

## Abstract

**Objectives:**

Using Jiangxi Province as a case study, this research aims to evaluate the progress in achieving the national “Healthy China 2030” all-cause health-adjusted life expectancy (HALE) targets at the subnational level.

**Methods:**

Based on provincial data collected between 2000 and 2020, we applied a simplified, validated model to estimate all-cause years lived with disability (YLD) rates. HALE was then calculated using the Sullivan method with provincial abridged life tables. Values for 2021–2030 were projected using the Lee-Carter and Autoregressive Integrated Moving Average (ARIMA) models. Missing data were handled via spline interpolation, and uncertainty was quantified via Monte Carlo simulation.

**Results:**

All-cause HALE in Jiangxi increased by 7.8 years from 2000 to 2020 and is projected to reach 71.6 years by 2030, meeting the national target despite a recent slowdown in growth. YLD rates declined in younger age groups but rose among those aged ≥80 years. Women consistently showed higher life expectancy and HALE than men, but accumulated more unhealthy life-years.

**Conclusions:**

Jiangxi Province is on track to achieve the “Healthy China 2030” HALE target. Persistent age and gender disparities highlight the need for tailored interventions—particularly for women and the growing elderly population—to improve health-related quality of life.

## Highlights

• Health-adjusted life expectancy in Jiangxi is projected to reach 71.6 years by 2030.
• A simplified model estimates disability burden from three provincial health indicators.
• Women live longer but spend more years in poor health than men.

## 1 Introduction

The shift from merely prolonging life to enhancing health-span is a central tenet of modern public health. Health-adjusted Life Expectancy (HALE), defined by the World Health Organization the average number of years a person can expect to live in full health, has thus emerged as a pivotal metric (World Health Organization, 2001). It integrates mortality and morbidity to offer a comprehensive assessment of population health, guiding global and national health policy-making and serving as a key indicator for monitoring Universal Health Coverage and the Sustainable Development Goals (World Health Organization, 2024; World Health Organization, 2025). Recognizing its value, the “Healthy China 2030” initiative has established HALE improvement as a core national policy target (The State Council of the People's Republic of China, 2025).

China's vast territory and large population, together with substantial inter-provincial disparities in socioeconomic development, mean that national-level health indicators cannot accurately reflect conditions in every region. In alignment with national policy directives, provincial governments have thus begun to develop localized HALE targets adapted to their specific contexts. As a result, understanding HALE at provincial and sub-provincial levels is especially critical for future health planning in a country of China's scale. Currently, research on all-cause HALE in China relies heavily on estimates from the Global Burden of Disease study(GBD) (Chen et al., 2022; Chen et al., 2019). However, the methodological complexity and high data demands of the GBD approach present significant limitations for its application (GBD 2021 Diseases and Injuries Collaborators, 2024; GBD 2019 Diseases and Injuries Collaborators, 2020).

The GBD study sets the standard for comprehensive HALE estimation but requires data-intensive micro-simulation, which is often unavailable at the subnational level in China. To address this gap, we developed a simplified model that captures the core relationship between key population health indicators and all-cause YLD rates, using China's GBD data as a reference. This study applies this pragmatic model to locally-sourced data from Jiangxi Province, aiming to generate a continuous, long-term series of HALE estimates that reflect local conditions and are actionable for provincial health planning.

## 2 Methods

### 2.1 Study design and population

This study provides a systematic estimation and projection of all-cause health-adjusted life expectancy at birth in Jiangxi Province, China, from 2000 to 2030 using aggregated, anonymized provincial-level data. No individual-level identifiable data were accessed.

### 2.2 Measures

(1) Life expectancy, 2000–2030
  Drawing on methodologies from the GBD study, we estimated under-five mortality (U5MR) and adult mortality rate in Jiangxi Province from 2000 to 2020 by constructing multiple linear regression models (Liu, 2022; Wilmoth et al., 2012; Schoen, 1978). Model parameters were based on the China Population Census (2000,2010,2020), 1% Population Sample Survey (2005, 2015), as well as the U5MR data of 2851 counties (cities/districts) in China published by GBD from 1996 to 2012. Covariates were obtained from the China Statistical Yearbook, provincial statistical yearbooks, and the National Bureau of Statistics database. We then applied the model life table method to calculate abridged life tables. Using age-specific mortality rates from 2000 to 2020, we estimated Lee–Carter parameters and projected age-specific mortality for 2021–2030, which were used to derive abridged life tables by sex (Schoen, 1978).
(2) Years lived with disability, 2000–2030
  In our previous work, we developed a simplified model for estimating all-cause YLD rates across 19 age-sex groups in China. This model was derived and validated using national-level data from the GBD study. When applied to China, the model-predicted all-cause YLD rates showed high consistency with GBD estimates, with a mean absolute error (MAE) of 0.0007 and a mean absolute percentage error (MAPE) of 0.5949%. Furthermore, the all-cause HALE calculated by combining these model outputs with GBD life tables demonstrated excellent agreement with GBD-reported HALE, with an MAE of 0.0341 years and a MAPE of 0.0526% (Chen et al., 2024). For application to Jiangxi Province, we used the regression coefficients (and their 95% uncertainty intervals) from the validated national model with locally sourced inputs. The model formula is specified as follows:
  $$\Gamma \left(Y_{i}\right)=\beta_{0i}+A_{i}X_{1}+B_{i}X_{2}+C_{i}X_{3}$$

where，$X_{1},X_{2},X_{3}$ are the incidence of statutorily reported class A/B infectious diseases (obtained from the China Health Statistics Yearbook), prevalence of chronic diseases among residents aged ≥15 years (derived from the Jiangxi component of the National Health Services Survey), and U5MR in Jiangxi Province (sourced from the Jiangxi Maternal and Child Surveillance System), respectively. $\beta_{0i}$ is the intercept, and$\;A_{i},B_{i},C_{i}$ are the regression coefficients of corresponding independent variables, respectively. Because an independent subnational years lived with disability dataset for Jiangxi was unavailable, external out-of-sample validation at the province level was not feasible; therefore, outputs are interpreted as a plausible, data-driven approximation of provincial trends.

Jiangxi Province's data on three key predictors are publicly available, aggregated, and anonymized provincial-level statistics. Given substantial missingness in the chronic disease prevalence series, missing years were imputed using quadratic spline interpolation, a method that assumes smooth, non-linear progression between observed points. To evaluate the robustness of this choice, a sensitivity analysis compared alternative interpolation approaches; the resulting variation in health-adjusted life expectancy estimates was minimal (maximum deviation ≤0.5 years), and all alternative estimates fell within the 95% uncertainty intervals of the primary analysis. Detailed preprocessing and sensitivity analysis are provided in the Appendix.

### 2.3 Statistical analysis

Based on the estimated all-cause YLD rates from 2000 to 2020, the Lee-Carter and Autoregressive Integrated Moving Average models were used to predict the all-cause YLD rates for the total population and for 19 age-sex groups in Jiangxi Province from 2021 to 2030. Uncertainty in YLD and HALE estimates was quantified via Monte Carlo simulation, which propagated uncertainty from national GBD YLD inputs through the provincial model. For methodological details, see the Appendix.

In this study, we used the life table technique and the Sullivan method to calculate all-cause HALE (Jagger et al., 2019). Trends in LE and YLD rates and HALE over years were analyzed by sex-segregated linear regression models, and the statistical significance of the trends was assessed based on the test of significance of the regression coefficients (*P* < 0.05). All analyses were performed using R software (version 4.1.0).

## 3 Results

### 3.1 Measurement of all-cause YLD rate in Jiangxi Province, 2000–2030

**Fig. 1:**
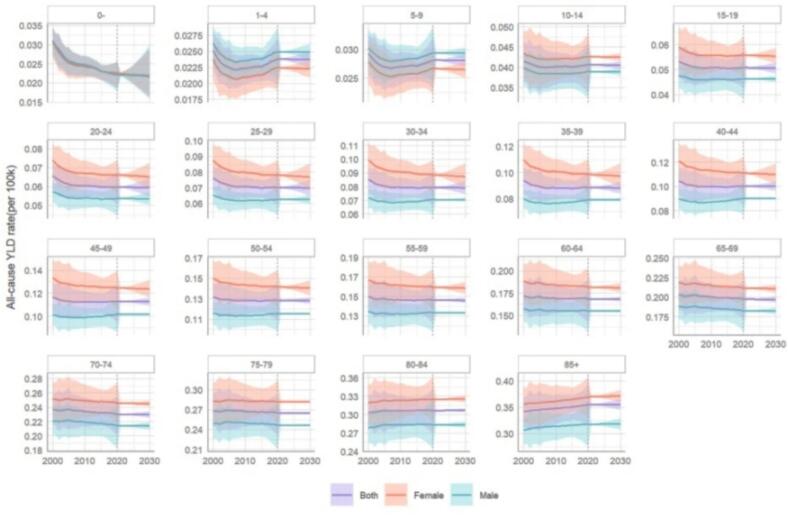
Age- and sex-specific trends in Years Lived with Disability rates in Jiangxi, China from 2000 to 2030. Note: Vertical dashed line indicates the start of the projection. Shaded areas represent 95% credible intervals. YLD=Years Lived with Disability; per 100 K = (/100,000).

Fig. 1 shows the all-cause YLD rates and their 95% uncertainty intervals for specific gender and age groups in Jiangxi Province, China, from 2000 to 2030.From 2000 to 2020, the YLD rates of the 0-year-old group declined the fastest (of which *P* < 0.01 for different genders), and the groups of 1–4 years old and 5–9 years old showed a decreasing and then increasing fluctuation without significant time trends, before and after a relatively. The YLD rate of 10–14, ……，75–79 years old group was generally decreasing (in which there was no significant time trend for males in some age groups, and *P* < 0.05 for females and both by age), and the YLD rate of 80 years old and above showed a significant upward trend (in which *P* < 0.01 for different genders). There was a strong positive correlation between all-cause YLD rates by sex and age. In terms of gender, the all-cause YLD rates of females and males were close to each other in the age group of less than 10 years, while the all-cause YLD rates of females were greater than those of males in the age group of 10 years and above, and the gap between the YLD rates of males and females was widening from the age of 15 years onwards.

### 3.2 Measurement of life expectancy (LE) in Jiangxi Province, 2000–2030

**Fig. 2:**
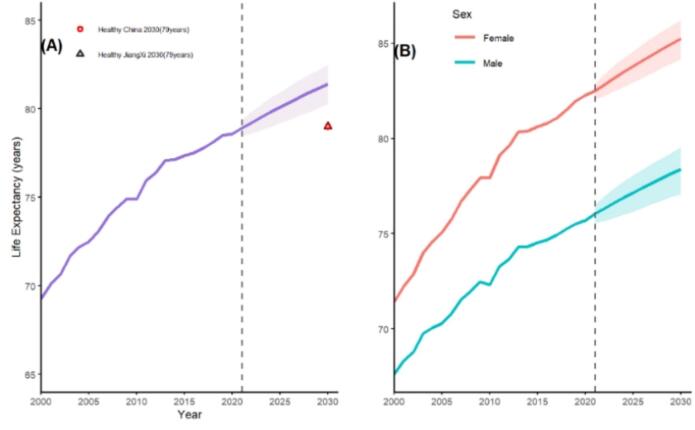
Trends and Projections of Life Expectancy in Jiangxi, China. Life expectancy for both sexes in Jiangxi, China. The solid purple line represents the life expectancy trend, and the light purple shaded area shows the predicted 95% credible interval. The red open circle indicates the life expectancy goal of the Healthy China 2030 Plan, and the black open triangle marks the life expectancy goal of the Healthy Jiangxi 2030 Plan. Life expectancy trends for females and males in Jiangxi, China. The solid light red line and solid blue line represent life expectancy for females and males, respectively, with the light red and light blue shaded areas indicating their corresponding 95% credible intervals. Note: The vertical dashed line denotes the start of the projection period. (For interpretation of the references to colour in this figure legend, the reader is referred to the web version of this article.)

Fig. 2 shows the trend and projection of life expectancy at birth (LE_0_) in Jiangxi, China. It can be seen from the figure that the LE in Jiangxi, China, increases significantly from 2000 to 2020 (*P* < 0.01), and it is expected that the growth of LE_0_ slows down in the latter 10 years. Figure 2 (A) shows the LE_0_ of the whole population in Jiangxi from 2000 to 2030, and ∆ represents the planning target of China and Jiangxi Province —79 years old in 2030.It can be found that the projected LE and the lower limit of its 95% confidence interval in Jiangxi, China in 2030 are higher than the planning targets of Healthy Jiangxi 2030 and Healthy China 2030. Figure 2 (B) shows the LE_0_ of men and women in Jiangxi from 2000 to 2030. It can be seen that the LE_0_ of the whole population, men and women in Jiangxi Province tends to increase over time, and the LE of females is always higher than that of males, and the gap between the two sexes is widening in 2021–2030.

### 3.3 Measurement of all-cause HALE in Jiangxi Province, 2000–2030

Table 1 shows the health-adjusted life expectancy at birth (HALE_0_) for different sexes in Jiangxi Province from 2000 to 2030. The results showed that the HALE_0_ of different genders increased steadily from 2000 to 2020 (*P* < 0.01 for different genders). In particular, the both HALE_0_ increased from 61.58 years (95% CI:61.03, 62.24) in 2000 to 69.39 years (95% CI:68.64, 70.34) in 2020, and from 62.24 years (95% CI:61.53, 63.03) in 2000 to 71.26 years (95% CI:70.51, 72.24) for females and from 60.99 years (95% CI: 60.61, 61.41) to 68.01 years (95% CI: 67.44, 68.73) for males. By 2030, HALE_0_ for the both is projected to increase to 71.55 years (95% CI:71.28, 71.84), an increase of only 1.9 years compared to 2021. Men will reach 70.13 years (95% CI:69.98, 70.29) and women 73.49 years (95% CI:73.00, 73.99). The HALE_0_ of women was consistently higher than that of men over the 21 years, with a difference of 1.25 years between the sexes in 2000 and 3.25 years higher in women than in men in 2020.By 2030, the gender gap is projected to be 3.36 years. The HALE_0_ gap between men and women is widening.

**Table 1:** Estimated values of Health-adjusted life expectancy at birth for specific sexes in Jiangxi Province from 2000 to 2030.

Year	Male	Female	Both
2000	60.99(60.61,61.41)	62.24(61.53,63.03)	61.58(61.03,62.24)
2001	61.66(61.29,62.08)	63.00(62.26,63.78)	62.30(61.76,62.98)
2002	62.09(61.58,62.68)	63.65(62.71,64.61)	62.84(62.05,63.68)
2003	62.94(62.52,63.41)	64.59(63.78,65.46)	63.71(63.11,64.42)
2004	63.22(62.78,63.69)	65.11(64.30,65.97)	64.15(63.51,64.86)
2005	63.42(62.89,63.97)	65.54(64.60,66.44)	64.43(63.69,65.21)
2006	63.91(63.50,64.40)	66.17(65.42,66.99)	64.96(64.35,65.64)
2007	64.55(64.13,65.04)	66.93(66.19,67.75)	65.63(65.05,66.33)
2008	64.89(64.50,65.37)	67.42(66.74,68.20)	66.05(65.51,66.73)
2009	65.30(64.94,65.75)	67.88(67.27,68.61)	66.46(65.96,67.08)
2010	65.21(64.85,65.66)	67.93(67.26,68.67)	66.45(65.92,67.08)
2011	65.98(65.63,66.43)	68.82(68.22,69.54)	67.28(66.79,67.91)
2012	66.29(65.95,66.72)	69.22(68.65,69.88)	67.63(67.17,68.22)
2013	66.82(66.51,67.25)	69.78(69.25,70.42)	68.16(67.75,68.75)
2014	66.85(66.56,67.28)	69.85(69.33,70.48)	68.22(67.82,68.78)
2015	67.01(66.72,67.44)	70.04(69.58,70.64)	68.40(68.01,68.94)
2016	67.13(66.84,67.59)	70.17(69.69,70.78)	68.53(68.14,69.12)
2017	67.31(66.98,67.81)	70.37(69.89,71.02)	68.72(68.25,69.39)
2018	67.54(67.12,68.08)	70.67(70.11,71.38)	68.98(68.44,69.72)
2019	67.76(67.27,68.36)	70.98(70.33,71.77)	69.24(68.61,70.09)
2020	68.01(67.44,68.73)	71.26(70.51,72.24)	69.39(68.64,70.34)
2021	68.24(68.23,68.24)	71.47(71.44,71.49)	69.65(69.64,69.67)
2022	68.50(68.48,68.52)	71.72(71.66,71.77)	69.89(69.86,69.92)
2023	68.72(68.69,68.75)	71.96(71.87,72.05)	70.12(70.07,70.16)
2024	68.94(68.90,68.98)	72.20(72.06,72.34)	70.34(70.27,70.42)
2025	69.15(69.09,69.21)	72.44(72.25,72.63)	70.56(70.45,70.67)
2026	69.36(69.29,69.43)	72.65(72.42,72.87)	70.77(70.64,70.89)
2027	69.56(69.46,69.64)	72.87(72.59,73.16)	70.98(70.81,71.13)
2028	69.75(69.64,69.87)	73.09(72.74,73.49)	71.18(70.98,71.40)
2029	69.94(69.82,70.07)	73.29(72.89,73.71)	71.37(71.14,71.60)
2030	70.13(69.98,70.29)	73.49(73.00,73.99)	71.55(71.28,71.84)

Note: Values in parentheses are 95% uncertainty intervals.

### 3.4 Ratio of HALE to LE

**Fig. 3:**
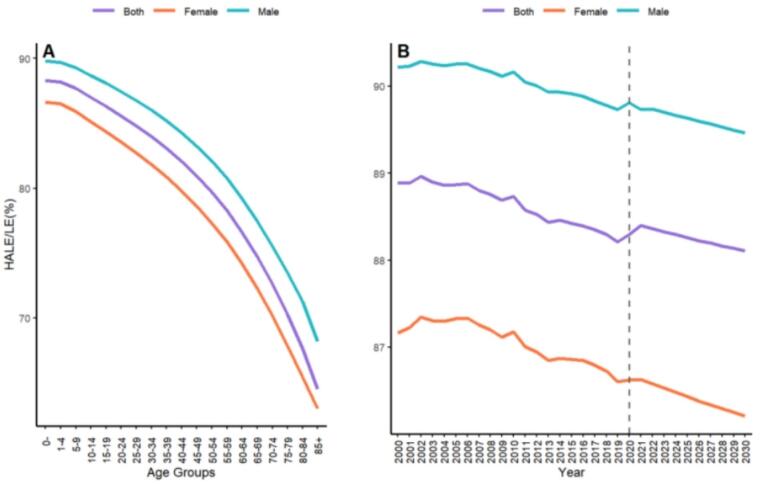
Ratio of age-specific Healthy-adjusted Life Expectancy to Life Expectancy for different genders in Jiangxi Province in 2020 (A); Changes in Healthy-adjusted Life Expectancy to Life Expectancy ratio for different genders in Jiangxi Province from 2000 to 2020 (B). Note: Vertical dashed line indicates the start of the projection. Three different colors represent three genders. HALE = Healthy Adjusted Life Expectancy; LE = Life Expectancy.

In Fig. 3A, which illustrates the ratio of HALE to LE across 19 age groups by sex in Jiangxi Province for 2020, it was observed that the ratio consistently decreases with advancing age for both sexes. Furthermore, the HALE-to-LE ratio for males remains consistently higher than that for females across all age groups. Fig. 3B reveals the trend in the HALE-to-LE ratio for the 0-year age group from 2000 to 2030. The ratio shows an overall declining trend over this period for both sexes, although the change is not pronounced.

## 4 Discussion

This study developed and applied a practical model to generate the first continuous, long-term series of HALE estimates for Jiangxi Province from 2000 to 2030. By leveraging simplified, locally-sourced data, we estimated trends in LE and HALE, projected future values, and analyzed age- and sex-specific disparities. Our core finding is a steady increase in both LE and HALE over the past two decades, with projections indicating that the overarching life expectancy target of the national “Healthy China 2030” and provincial “Healthy Jiangxi 2030” initiatives is on track to be met. It is important to emphasize that, as a descriptive trend analysis, this work primarily provides a monitoring framework and a macro-level assessment of progress toward broad policy objectives, rather than a causal evaluation of specific interventions.

The all-cause YLD rate is an important indicator for quantifying non-fatal health consequences (GBD 2019 Diseases and Injuries Collaborators, 2020). From 2000 to 2020, the all-cause YLD rate in Jiangxi Province showed a declining trend, which is consistent with the trend observed in the GBD estimates for China from 1990 to 2021 (GBD 2021 Diseases and Injuries Collaborators, 2024). The observed decline in the all-cause YLD rate in Jiangxi Province occurred alongside a period of significant investment and effort to strengthen the primary healthcare system, notably marked by improvements in the accessibility and quality of medical resources in rural areas. This period also saw dedicated initiatives in infectious disease control, chronic disease management, and health education, which are plausibly associated with a reduction in the incidence and disability rates of certain conditions (Chen et al., 2022; Wang et al., 2022). For example, the expansion of early screening and management for chronic diseases such as hypertension and diabetes aligns with strategies known to prevent complications (Chen et al., 2022; Nanchang Municipal People‘s Government, 2025). Furthermore, the “Healthy China 2030” strategy may have been a key driver in reforming and developing local healthcare systems (Jiangxi Provincial Health Commission, 2025; National Health Commission of the People‘s Republic of China, 2025).

Between 2000 and 2020, both life LE and HALE in Jiangxi Province showed steady growth. Over these 21 years, Jiangxi, China achieved an LE increase of 9.31 years (averaging 0.443 years annually) and HALE increase of 7.81 years (averaging 0.372 years annually). These gains align with nationwide upward trends documented in GBD-based studies (Chen et al., 2022). By 2020, Jiangxi's HALE reached 69.39 years, surpassing both the national average (68.50 years) and the global average (63.30 years) (World Health Organization, 2021), yet remaining below that of more developed regions such as Chongqing Municipality (71.70 years in 2017) (Ruan et al., 2021). These comparisons indicate that while Jiangxi performs well relative to broader benchmarks, further progress is possible. Research indicates that reducing the U5MR is one of the key drivers for increasing LE (Kefale et al., 2025) and the decline in all-cause YLD, has directly supported the rise in HALE.

The planning target of “Healthy China 2030”/“Healthy Jiangxi 2030” is to reach 79.0 years of LE per capita in 2030, with significant/gradual increase in HALE per capita (The State Council of the People's Republic of China, 2025; Jiangxi Provincial Health Commission, 2025). The results of this study show that by 2030, the LE for the total population of Jiangxi Province is projected to reach 81.4 years, exceeding both the national target for China and Jiangxi's provincial target of 79 years. The per capita HALE is expected to reach 71.55 years, representing an increase of nearly 10 years compared to the HALE in 2000, which will enable the achievement of the LE and HALE targets set by both “Healthy Jiangxi” and “Healthy China” initiatives. From 2021 to 2030, LE is projected to increase by an average of 0.249 years annually, while HALE is expected to grow by 0.19 years per year. However, the growth rates of both LE and HALE have slowed significantly compared to the previous two decades. This deceleration is associated with global trends of population aging, as the higher prevalence of chronic diseases among elderly populations has exerted a negative impact on HALE growth (Zhou et al., 2019; Xi et al., 2025). Furthermore, the law of diminishing marginal returns in health gains suggests that: During early stages of economic development, improvements in medical conditions and implementation of public health policies significantly boost both LE and HALE. However, as economic development progresses, the difficulty of achieving further health improvements increases, resulting in a slower growth rate of LE and HALE (Hensher et al., 2024).

The findings of this study demonstrate that while females exhibit higher HALE than males, their ratio of HALE to LE is comparatively lower. This observation aligns consistently with research findings from both domestic and international studies (Chen et al., 2022; Oksuzyan et al., 2010). This suggests that women experience a relatively longer period of unhealthy status throughout their life course, with lower overall health quality compared to men. The study's findings on all-cause YLD rates - showing similar values between genders before age 10 but significantly higher rates for women after age 10 - further corroborate this observation. Some scholars have termed this phenomenon the “male-female health-survival paradox,” attributing it to multiple potential factors (Alberts et al., 2014). Although women enjoy a longevity advantage, this is largely attributable to biological factors. However, socioeconomic and gender inequalities in areas such as educational attainment and employment conditions make women more likely to experience cumulative disadvantages throughout their life course (Farrelly, 2023). Additionally, from sociological and psychological perspectives, women demonstrate comparatively poorer performance than men in health-seeking behaviors such as voluntary healthcare utilization and adherence to medical advice (Borchers and Gershwin, 2012). This finding underscores the need for targeted interventions to mitigate gender-based disparities in health quality and narrow the life-quality gap between sexes.

The finding that the ratio of HALE to LE decreases with age may be due to the general pattern of a natural tendency for individual health to deteriorate with age, as well as the fact that newborns are generally in better health and have access to more efficient preventive health measures. For older adults, early diagnosis and treatment of chronic disease is key to reducing the burden of all-cause YLD and improving quality of life and thus elevating HALE (Nature Editorial, 2025). From 2000 to 2020, the ratio of HALE to LE in Jiangxi Province showed a modest decline of approximately 0.5%. This trend suggests that while challenges remain, Jiangxi has made substantial progress in improving population health quality, demonstrating the effectiveness of its health policies. These results provide an optimistic outlook for future.

This study has several key limitations. First, the estimated all-cause HALE for Jiangxi Province does not account for the morbidity and mortality impacts of the COVID-19 pandemic, as this period represented an atypical global health shock. However, existing research suggests that the pandemic's effect on YLD rates and mortality in China was relatively limited (Liu et al., 2021). During the initial phase of the outbreak, China's stringent containment measures effectively curtailed transmission, resulting in a comparatively modest impact on Jiangxi Province (Abbas et al., 2021). Nonetheless, estimates for all-cause HALE for the years 2019–2022 may still contain some degree of bias. Second, and most important, the all-cause YLD model cannot disentangle the contributions of specific diseases, injuries, or risk factors (e.g., nutritional risks, hypertension, or diabetes) to changes in HALE, limiting actionable insights for targeted interventions. Third, the ecological and descriptive design precludes causal inference or control for confounding. Finally, while sensitivity analyses indicated robustness, the high degree of data imputation for key inputs remains a source of potential error. Future work should aim to decompose HALE by cause and risk factor to inform more precise policy.

## 5 Conclusion

This study systematically evaluated the healthy China/Jiangxi 2030 target of life expectancy in Jiangxi province, which increased by 7.8 years from 2000 to 2030 (71.6 years in 2030). Although the growth rate has slowed down, the goal of “Healthy China/Jiangxi 2030” will still be achieved. While infant/child YLD rates declined significantly, elderly populations face rising burdens, and gender disparities persist—women exhibit longer LE/HALE but accumulate more unhealthy life years. These findings mandate targeted interventions: prioritizing women's lifelong health access and strengthening geriatric care to address aging-related chronic diseases, ensuring equitable health gains across all demographics.

## Clinical trial number

Not applicable

## CRediT authorship contribution statement

**Jing Ding:** Writing – review & editing, Writing – original draft, Conceptualization, Formal analysis. **Sanqian Chen:** Writing – review & editing, Data curation, Investigation, Validation. **Yuting Deng:** Formal analysis, Visualization, Writing – review & editing. **Siyu Zhou:** Visualization, Data curation, Writing – review & editing. **Zhang Zeng:** Funding acquisition, Resources, Writing – review & editing. **Songbo Hu:** Supervision, Funding acquisition, Conceptualization, Methodology, Project administration, Writing – review & editing.

## Consent for publication

All the authors have approved the content of the submitted manuscript and understand the text and data of this manuscript will be freely available to the general public once it gets accepted.

## Ethics approval and consent to participate

Not applicable. The incidence of infectious diseases in Jiangxi province was from China Health Statistics Yearbook. The prevalence rate of chronic diseases in people over 15 years old was obtained from the six China Health Service surveys in Jiangxi province, and the mortality rate of children under 5 years old was obtained from the maternal and child surveillance data. These two data were internal data and not publicly released, provided by the Jiangxi Health Development Center.

## Funding

This study was partially supported by grants from the Natural Science Foundation of Jiangxi Province (Grant No. 20224BAB206094) and the National Natural Science Foundation of China (Grant No. 81960618).

## Declaration of competing interest

The authors declare that they have no known competing financial interests or personal relationships that could have appeared to influence the work reported in this paper.

## Data Availability

Data will be made available on request.

## Appendix A Supplementary data

Supplementary data: This supplementary material provides detailed information on the abbreviated life table for Jiangxi Province, covariate data processing and imputation methods, sensitivity analysis for interpolation robustness, uncertainty analysis using Monte Carlo simulation, and projections of all-cause Years Lived with Disability (YLD) rates for 2021–2030. Supplementary Tables S1–S3 present comparisons of imputation methods for chronic disease prevalence, under-five mortality rate, and their impact on healthy-adjusted life expectancy (HALE) estimates.Thank you for your assistance. Image 1
