# Supplementary material for: All-cause health-adjusted life expectancy in Jiangxi Province, China, 2000–2030: systematic estimation and analysis
Source: Prev Med Rep. 2026 Feb 28;64:103436. doi: 10.1016/j.pmedr.2026.103436 (PMC12972705; doi:10.1016/j.pmedr.2026.103436)
Supplement: Supplementary file 1 — Supplementary data: This supplementary material provides detailed information on the abbreviated life table for Jiangxi Province, covariate data processing and imputation methods, sensitivity analysis for interpolation robustness, uncertainty analysis using Monte Carlo simulation, and projections of all-cause Years Lived with Disability (YLD) rates for 2021–2030. Supplementary Tables S1–S3 present comparisons of imputation methods for chronic disease prevalence, under-five mortality rate, and their impact on healthy-adjusted life expectancy (HALE) estimates.Thank you for your assistance. [file mmc1.docx]

Supplementary appendix

**Content**

[**1 Introduction to the Abbreviated Life Expectancy Table for Jiangxi Province** 2](#_Toc222949246)

[**2 Principles and applications of all-cause Years Lived with Disability rate models** 3](#_Toc222949247)

[(1) Incidence of Class A and B Notifiable Infectious Diseases in Jiangxi Province 3](#_Toc222949248)

[(2) Chronic disease prevalence rate among population aged 15+ in Jiangxi Province 3](#_Toc222949249)

[(3) Under-five mortality rate in Jiangxi province 4](#_Toc222949250)

[**3 Uncertainty Analysis (Monte Carlo Simulation)** 6](#_Toc222949251)

[**3.2 Input Distribution Assumptions** 6](#_Toc222949252)

[**3.3 Modeling Age-Group Correlation** 7](#_Toc222949253)

[**3.4 Simulation Implementation** 7](#_Toc222949254)

[**4 Projections of all-cause Years Lived with Disability rates in Jiangxi Province, 2021-2030** 8](#_Toc222949255)

[**5 References** 10](#_Toc222949256)

[TableS1 Comparison of different interpolation methods for the prevalence of chronic diseases among people over 15 years old 11](#_Toc222949257)

[TableS2 Comparison of different interpolation methods for mortality rates of children under 5 years old 11](#_Toc222949258)

[TableS3 Impact of Different Interpolation Methods on the Estimated Healthy-adjusted Life Expectancy in 2020 12](#_Toc222949259)

**1 Introduction to the Abbreviated Life Expectancy Table for Jiangxi Province**

This study measured abbreviated life tables for men and women in Jiangxi Province from 2000-2020, with data and methods cited from one of our previous studies. In this study Liu measured the life tables of Jiangxi Province for 2000-2019 using the model life table method, and predicted the gender-specific life tables of Jiangxi Province for 2020-2030 using the Lee-Carter model, stochastic extrapolation model combined with the Jiang-Qing Luang method. At the same time, the paper also used the current life expectancy data of each province published in the Statistical Yearbook as the real reference value to evaluate the error of the model, and the results showed that the Mean Absolute Error (MAE), Mean Absolute Percentage Error (MAPE), Root Mean Square Error (RMSE) are all less than 2% (when it is lower than 10%, it can be considered that the estimation accuracy is higher). Root Mean Square Error, RMSE) were all less than 2% (when less than 10%, the estimation accuracy can be considered high). For more detailed information on estimation, see his paper (1). This study only calculated the life expectancy for males and females in Jiangxi Province from 2000 to 2020, and did not compute abridged life tables for the total population. We used age-specific mortality rates for males and females from 2000 to 2020, combined with age-specific population data, to derive the age-specific mortality rates for the total population during the same period. The calculation was performed using the following formula: (Mortality rate of a specific age group for males$\times$Total male population in the corresponding age group$+$ Mortality rate of the corresponding age group for females $\times$ Total female population in the corresponding age group) / Total population of the corresponding age group. Subsequently, we applied Chiang’s method to compute the abridged life tables for 2000–2020 (2).

**2 Principles and applications of all-cause Years Lived with Disability rate models**

**2.1** **Methods for Handling Covariates Prior to Model Application**

(1) Incidence of Class A and B Notifiable Infectious Diseases in Jiangxi Province

Since the China Statistical Yearbook only published the incidence rates of legally reported infectious diseases of category A and B in Jiangxi Province from 2002 to 2020, this study utilized the literature search method to fill in the data for 2000 and 2001. We searched major Chinese and English databases using keywords such as "infectious diseases," "incidence rate," and "Jiangxi," and obtained the 2000-2001 incidence rates of Class A and B notifiable infectious diseases for Ganzhou City(3), Jiujiang City (4) and Nanchang County (5). By combining these rates with population data, we calculated a composite rate for these three areas, which was then used as a proxy for the incidence rate of Class A and B notifiable infectious diseases in Jiangxi Province for 2000 and 2001.

(2) Chronic disease prevalence rate among population aged 15+ in Jiangxi Province

Since the Jiangxi Health Development Center only provided chronic disease prevalence rates for the population aged 15+ in 2003, 2008, 2013, and 2018, we used spline interpolation based on these three data points to estimate values for the missing years. Consequently, data points for 17 out of 21 years (81.0%) required imputation. To generate a complete annual series, we employed quadratic spline interpolation as the primary method. This approach assumes a smooth, non-linear trend between the three observed data points. The *spline* function from the *splines* package in R was used to produce smoothed estimates for all years from 2000 to 2020. The same procedure was applied independently to estimate gender-specific (male and female) prevalence rates.

(3) Under-five mortality rate in Jiangxi province

In the same case, only the under-5 mortality rate for 2005-2020 is published on the “Summary of Jiangxi Province's Health and Wellness Statistics”, and we obtained the number of deaths and the total population by age from the information of the Fifth National Population Census, and calculated the under-5 mortality rate according to the formula "deaths at the age of 0 years + deaths at the age of 1-4 years / total population at the age of 0 years + total population at the age of 1-4 years " to calculate the under-five mortality rate in 2000. Missing data were then filled in using spline interpolation based on the under-5 mortality rates for 2000 and 2005-2020. Therefore, only the data for four years (2001–2004, 19.0% of the series) were missing and required imputation. We applied quadratic spline interpolation to fill the 2001–2004 gap, using the known values for 2000 and 2005–2020 as anchor points. This was implemented using the spline function in R.

**2.2 Sensitivity Analysis for Imputation Robustness**

1. Methods of Sensitivity Analysis

To assess the robustness of our results to the choice of imputation technique, we re-estimated the missing chronic disease prevalence data using five alternative methods: Linear interpolation 、Cubic spline interpolation、Last observation carried forward (LOCF) 、Exponential growth and Quadratic polynomial；For U5MR, we conducted a parallel sensitivity analysis using four alternative imputation strategies for the 2001-2004 period: Linear interpolation、Moving Average Method (window=2)、Exponential decay and quadratic_polynomial_u5mr .

1. Results of Sensitivity Analysis

The sensitivity analysis comparing multiple imputation methods demonstrated that our core findings are robust to the specific choice of interpolation technique:

1) Chronic Disease Prevalence Rate among Population Aged 15+ (85.7% missing data)

As shown in Supplementary Table S1, six imputation methods were compared. All methods reproduced the three known data points (2003, 2008, 2013, 2018). The quadratic spline (primary method) yielded a mean prevalence of 0.256 (SD=0.125) over the study period. The mean absolute difference from this primary estimate was smallest for the exponential growth model (0.0027) and cubic spline/quadratic polynomial (0.0041), and largest for the LOCF method (0.0619). The maximum absolute difference for any single year was 0.150 (LOCF method, 2000-2002). The linear interpolation method, often considered a conservative alternative, differed by an average of only 0.0073.

2) Under-five mortality rate in Jiangxi province (19.0% missing data)

Five methods were compared (Supplementary Table S2). The primary quadratic spline method yielded a mean U5MR of 0.0197 (SD=0.0115). Differences between all methods were minimal, with mean absolute differences ranging from 0.00035 (linear) to 0.00191 (quadratic polynomial). The maximum absolute difference for any single imputed year (2001-2004) was 0.00932.

3) Impact of Different Imputation Methods on Healthy-adjusted Life Expectancy Estimates for the Year 2020

The choice of imputation method for chronic disease prevalence (81% missing) accounts for nearly all the observed variation in final HALE estimates. The LOCF method produced the largest deviation, increasing HALE estimates by approximately 0.23–0.31 years across the study period. The imputation method for the under-5 mortality rate (19% missing) had an almost undetectable effect on HALE, with differences not exceeding 0.01 years. Even the largest deviation (LOCF, +0.31 years in 2020) is substantially smaller than the width of the reported 95% uncertainty intervals for HALE (approximately 1.5–2.0 years) (Supplementary Table S3). All alternative method results fall well within the primary analysis's uncertainty bounds.

**3 Uncertainty Analysis (Monte Carlo Simulation)**

**3.1 Sources of Uncertainty Propagated**

The analysis aimed to propagate uncertainty originating from the GBD's national-level all-cause YLD rate estimates through our model to the final provincial HALE estimates. This encompasses uncertainty in the GBD input data, the derived model coefficients ($\beta_{0},\beta_{1},\beta_{2},\beta_{3}$), and the final outputs.

**3.2 Input Distribution Assumptions**

We assumed that the GBD YLD rate for each age-year group follows a normal distribution. The mean ($\mu$) was the GBD point estimate. The standard deviation ($\sigma$) was derived from the reported GBD 95% uncertainty intervals using the formula: $\sigma\approx(UI_{upper}-UI_{lower})/(2\times1.96)$.

**3.3 Modeling Age-Group Correlation**

To preserve the correlation structure between age groups, we modeled the 19 age-specific YLD rates for each year using a 19-dimensional multivariate normal distribution. The mean vector was the set of GBD point estimates for that year. The covariance matrix was constructed by first calculating the sample covariance matrix from the 30-year (1990-2019) national GBD time series to capture the correlation pattern between age groups. We assumed this correlation structure remained constant. For each specific year, the diagonal of this matrix (variances) was replaced with the year-specific variances calculated as described above.

**3.4 Simulation Implementation**

For each year (1990-2019), we drew 1,000 independent random samples from its respective multivariate normal distribution using the mvrnorm function in R. This sample size ensured stable distributions for our output metrics. Each of the 1,000 simulated national YLD datasets was used to refit the model, producing 1,000 sets of coefficients. Each coefficient set was then applied with Jiangxi's local inputs to produce 1,000 provincial YLD and HALE trajectories. The 2.5th and 97.5th percentiles of the resulting 1,000 values for any given metric (e.g., HALE in 2030) define its 95% uncertainty interval.

**4 Projections of all-cause Years Lived with Disability rates in Jiangxi Province, 2021-2030**

This study used the Lee-Carter model to predict age-specific all-cause YLD rates in a two-step process:

Step 1: Singular value decomposition method to solve the three model parameters Three parameters, $\alpha_{x}$，$\beta_{x}$，$\kappa_{t}$ , were estimated from 2000 to 2020 using the all-cause YLD rates of the whole population, males, and females in 19 age groups in Jiangxi Province from 2000 to 2020 as the basis.

The three-parameter estimation process in step 1 was implemented in R language software, with code from the official website of the demographic methodology established by Prof. Germán Rodríguez at Princeton University.（[https://grodri.github.io/demography/leecarter#R](file:///C:\Users\PC\Documents\WPS%20Cloud%20Files\.416686369\cachedata\88053F42A5E34E13B3CEA4E8B48DA05C\2000-2030年江西省ACHLE的测算.docx#R)）。

Step 2: The Autoregressive Integrated Moving Average (ARIMA) predicts $\kappa_{t}$。

Using the above estimated $\kappa_{t}$for 2000−2020 as a time series, ARIMA is utilized to predict$\kappa_{t}$ARIMA (2, 2, 0) is used in this study. The YLD rate for 2021−2030 is then calculated based on the following equation:

$Y_{x,s+t}={exp(\alpha}_{x}+\beta_{x}\kappa_{s+t})$ （1）

In the above equation, $\kappa_{s+t}$is the predicted value of the parameter$\kappa_{t}$at the moment of $s+t$， $\alpha_{x}$and$\beta_{x}$are the estimates at the age of $x$ , which do not vary with the year. The Lee-Carter model is essentially an extrapolation model, and in order to improve the accuracy of the model's prediction, it is usually extrapolated with the YLD rate at the last observed year $s$ (6) , which is given in the following equation:

$Y_{x,s+t}=Y_{x,s}\times{exp(\beta}_{x}(\kappa_{s+t}-\kappa_{s})), s<s+t$ （2）

This study also extrapolates according to equation (2), where year $s$is 2020.

**5 References**

1. Liu Z. A study on the calculation of life expectancy by gender in Jiangxi Province from 2000 to 2030 [Master‘s thesis] (江西省2000-2030年分性别预期寿命测算研究[硕士学位论文]). [cited 2025 May 21]. Available from: https://doi.org/10.27232/d.cnki.gnchu.2022.000603.
2. Deng HZ, Wu ZF, Li R. Surveillance and analysis of legally notifiable infectious diseases in Ganzhou City, 2000-2006 [Internet]. Journal of Gannan Medical University. 2008 [cited 2025 Apr 7];(4):546–548, 550. Available from: <https://kns.cnki.net/KCMS/detail/detail.aspx?dbcode=CJFQ&dbname=CJFD2008&filename=GNYX200804035>
3. Cheng LJ, Wu JC, Huang CG. Epidemiological trends of legally notifiable infectious diseases in Jiujiang City, 1997-2006 [Internet]. Practical Preventive Medicine. 2008 [cited 2025 Apr 7];(2):437–439. Available from: <https://kns.cnki.net/KCMS/detail/detail.aspx?dbcode=CJFQ&dbname=CJFD2008&filename=SYYY200802053>
4. Zhu HW, You XH. Dynamic analysis of legally reported infectious diseases in Nanchang County, 1994-2003 [Internet]. South China Journal of Preventive Medicine. 2004 [cited 2025 Apr 7];(5):15–16. Available from: <https://kns.cnki.net/KCMS/detail/detail.aspx?dbcode=CJFQ&dbname=CJFD2004&filename=GDWF200405006>
5. Pitacco E, Denuit M, Haberman S, Olivieri A. Modelling longevity dynamics for pensions and annuity business [Internet]. Oxford University Press; 2009 [cited 2025 Jul 3]. Available from: https://doi.org/10.1093/oso/9780199547272.001.0001

TableS1 Comparison of different interpolation methods for the prevalence of chronic diseases among people over 15 years old

| Method | Mean | SD | Min | Max | Mean_Abs_Diff | Max_Abs_Diff |
| --- | --- | --- | --- | --- | --- | --- |
| Quadratic spline (primary) | 0.25589 | 0.12503 | 0.09343 | 0.4976 | 0.00000 | 0.00000 |
| Linear spline | 0.26307 | 0.09443 | 0.12956 | 0.43583 | 0.00726 | 0.01608 |
| Cubic spline | 0.25629 | 0.12469 | 0.11353 | 0.50513 | 0.00410 | 0.02010 |
| LOCF | 0.30894 | 0.11053 | 0.12956 | 0.43583 | 0.06188 | 0.15022 |
| Exponential growth | 0.25688 | 0.12600 | 0.10171 | 0.51314 | 0.00269 | 0.01554 |
| Quadratic polynomial | 0.25629 | 0.12469 | 0.11353 | 0.50513 | 0.00410 | 0.02010 |

*Note: Mean_Abs_Diff and Max_Abs_Diff are calculated as the average and maximum absolute differences from the primary (quadratic spline) method across all years (2000-2020). LOCF = Last Observation Carried Forward. *

TableS2 Comparison of different interpolation methods for mortality rates of children under-five years old

| Method | Mean | SD | Min | Max | Mean_Abs_Diff | Max_Abs_Diff |
| --- | --- | --- | --- | --- | --- | --- |
| Quadratic spline (primary) | 0.01970 | 0.01151 | 0.00660 | 0.04807 | 0.00000 | 0.00000 |
| Linear spline | 0.02005 | 0.01195 | 0.00660 | 0.04807 | 0.00035 | 0.00232 |
| Moving average | 0.01832 | 0.00997 | 0.00660 | 0.04807 | 0.00139 | 0.00932 |
| Exponential decay model | 0.01951 | 0.01066 | 0.00680 | 0.04174 | 0.00139 | 0.00633 |
| quadratic_polynomial_u5mr | 0.01996 | 0.01149 | 0.00835 | 0.04393 | 0.00191 | 0.00419 |

*Note: Mean_Abs_Diff and Max_Abs_Diff are calculated as the average and maximum absolute differences from the primary (quadratic spline) method across all years (2000-2020). The moving average method used a window of two surrounding years. U5MR= Under-five Mortality *

TableS3 Impact of Different Interpolation Methods on the Estimated Healthy-adjusted Life Expectancy in 2020

| Scenario | HALE in 2020 (years) | Absolute Difference from Primary Analysis (years) |
| --- | --- | --- |
| Primary Analysis (Baseline) | 69.39 | reference |
| Chronic Disease Prevalence: Linear Interpolation Only | 69.44 | 0.05 |
| Chronic Disease Prevalence: Cubic Spline Interpolation Only | 69.40 | 0.01 |
| Chronic Disease Prevalence: LOCF Method Only | 69.70 | 0.31 |
| Chronic Disease Prevalence: Exponential Growth Model Only | 69.38 | -0.01 |
| U5MR: Linear Interpolation Only | 69.39 | 0.00 |
| U5MR: Moving Average Method Only | 69.38 | -0.01 |
| Most Extreme Scenario (LOCF for Chronic Disease & Moving Average for U5MR) | 69.69 | 0.30 |

*Notes: HALE values are in years. Differences are calculated as (Alternative Method HALE - Primary HALE). All differences are ≤ 0.01 years, indicating a negligible impact from U5MR imputation method choice. HALE = Healthy-adjusted Life Expectancy; LOCF = Last Observation Carried Forward; U5MR= Under-5 Mortality *
